# Supplementary material for: Breeding range shift of the red-crowned crane (Grus japonensis) under climate change
Source: PLoS One. 2020 Mar 12;15(3):e0229984. doi: 10.1371/journal.pone.0229984 (PMC7067427; doi:10.1371/journal.pone.0229984)
Supplement: S2 Fig — Blue polygons are the current actual breeding area, green polygons are the predicted potential breeding area under current climate condition, and orange areas are the predicted potential breeding area climate change. Scenario is RCP6.0, intermediate emission scenario. (DOC) [file pone.0229984.s002.doc]

**
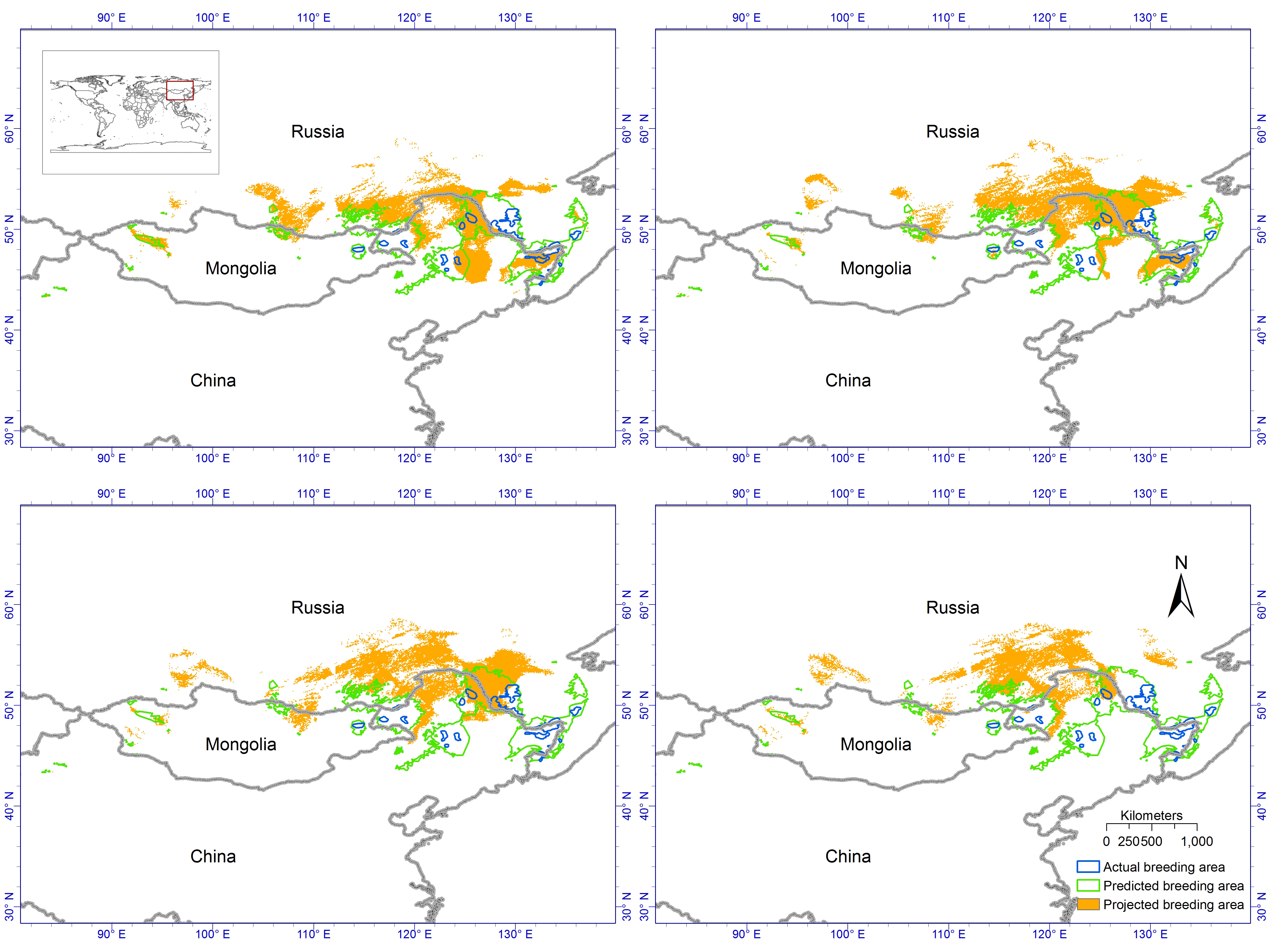
**

**S2 Fig. Predicted breeding range of the continental population of the red-crowned crane at 2030s, 2050s, 2070s and 2080s.** Blue polygons are the current actual breeding area, green polygons are the predicted potential breeding area under current climate condition, and orange areas are the predicted potential breeding area climate change. Scenario is RCP6.0, intermediate emission scenario.
